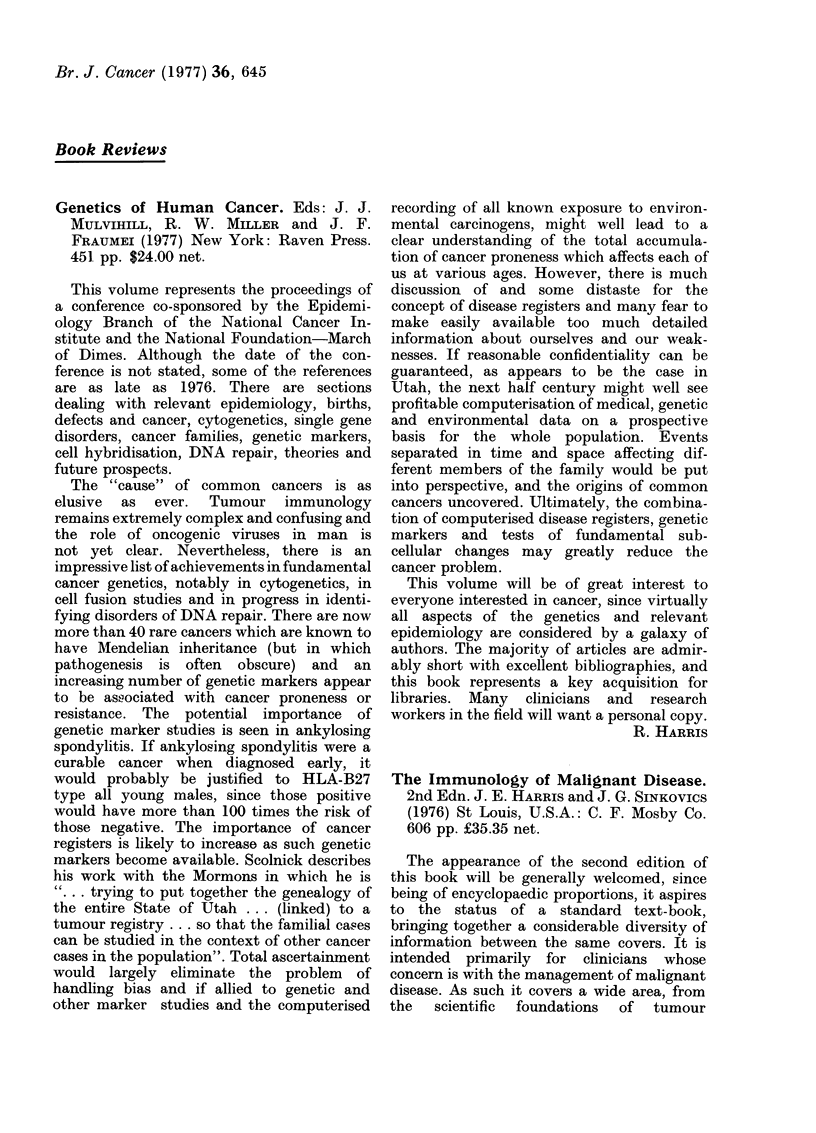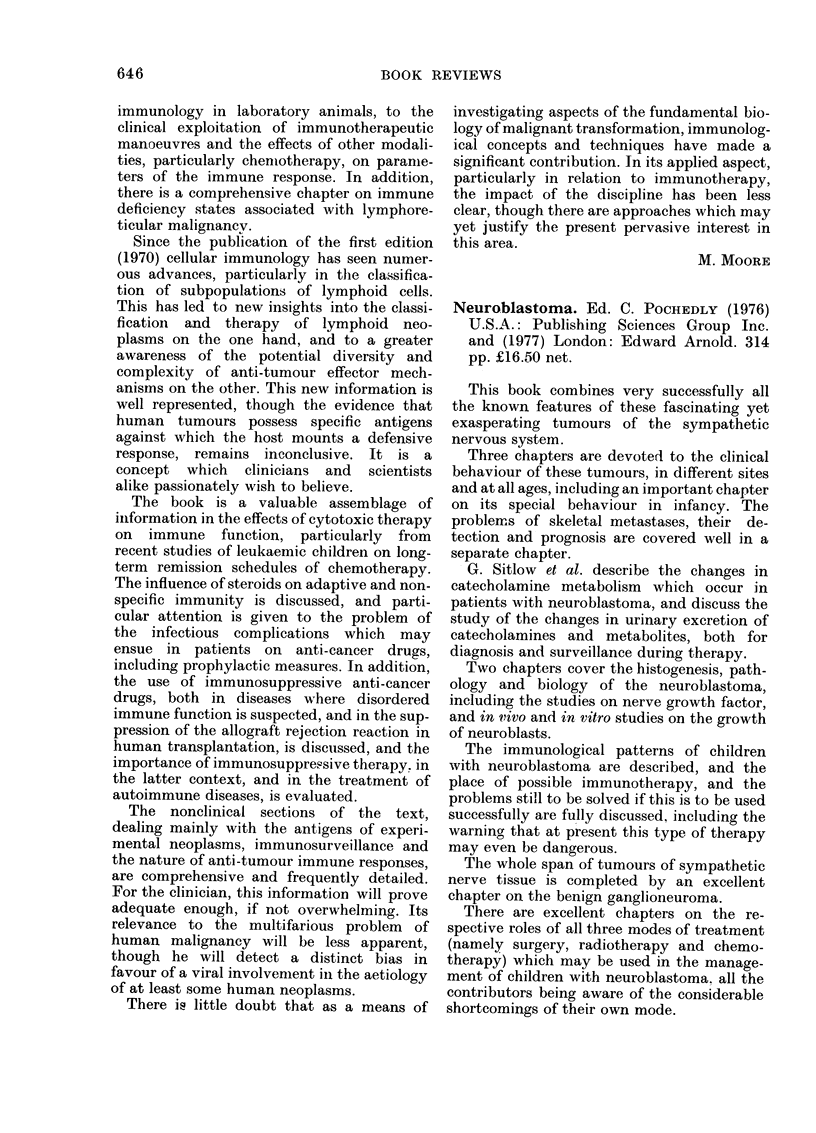# The Immunology of Malignant Disease

**Published:** 1977-11

**Authors:** M. Moore


**The Immunology of Malignant Disease.** 2nd Edn. J. E. Harris and J. G. Sinkovics (1976) St Louis, U.S.A.: C. F. Mosby Co. 606 pp. £35.35 net.

The appearance of the second edition of this book will be generally welcomed, since being of encyclopaedic proportions, it aspires to the status of a standard text-book, bringing together a considerable diversity of information between the same covers. It is intended primarily for clinicians whose concern is with the management of malignant disease. As such it covers a wide area, from the scientific foundations of tumour immunology in laboratory animals, to the clinical exploitation of immunotherapeutic manoeuvres and the effects of other modalities, particularly chemotherapy, on parameters of the immune response. In addition, there is a comprehensive chapter on immune deficiency states associated with lymphoreticular malignancy.

Since the publication of the first edition (1970) cellular immunology has seen numerous advances, particularly in the classification of subpopulations of lymphoid cells. This has led to new insights into the classification and therapy of lymphoid neoplasms on the one hand, and to a greater awareness of the potential diversity and complexity of anti-tumour effector mechanisms on the other. This new information is well represented, though the evidence that human tumours possess specific antigens against which the host mounts a defensive response, remains inconclusive. It is a concept which clinicians and scientists alike passionately wish to believe.

The book is a valuable assemblage of information in the effects of cytotoxic therapy on immune function, particularly from recent studies of leukaemic children on long-term remission schedules of chemotherapy. The influence of steroids on adaptive and non-specific immunity is discussed, and particular attention is given to the problem of the infectious complications which may ensue in patients on anti-cancer drugs, including prophylactic measures. In addition, the use of immunosuppressive anti-cancer drugs, both in diseases where disordered immune function is suspected, and in the suppression of the allograft rejection reaction in human transplantation, is discussed, and the importance of immunosuppressive therapy, in the latter context, and in the treatment of autoimmune diseases, is evaluated.

The nonclinical sections of the text, dealing mainly with the antigens of experimental neoplasms, immunosurveillance and the nature of anti-tumour immune responses, are comprehensive and frequently detailed. For the clinician, this information will prove adequate enough, if not overwhelming. Its relevance to the multifarious problem of human malignancy will be less apparent, though he will detect a distinct bias in favour of a viral involvement in the aetiology of at least some human neoplasms.

There is little doubt that as a means of investigating aspects of the fundamental biology of malignant transformation, immunological concepts and techniques have made a significant contribution. In its applied aspect, particularly in relation to immunotherapy, the impact of the discipline has been less clear, though there are approaches which may yet justify the present pervasive interest in this area.

M. MOORE